# Native collagen VI delays early muscle stem cell differentiation

**DOI:** 10.1242/jcs.261419

**Published:** 2024-02-12

**Authors:** Samuele Metti, Francesco Da Ros, Giorgia Toniato, Matilde Cescon, Paolo Bonaldo

**Affiliations:** Department of Molecular Medicine, University of Padova, 35131 Padova, Italy

**Keywords:** Extracellular matrix, Skeletal muscle, Muscle stem cells, Collagen VI

## Abstract

Adult muscle stem cells (MuSCs) are critical for muscle homeostasis and regeneration, and their behavior relies on a finely regulated niche made of specific extracellular matrix (ECM) components and soluble factors. Among ECM proteins, collagen VI (Col6) influences the mechanical properties of the niche and, in turn, MuSC self-renewal capabilities. Here, we investigated whether Col6 can exert a direct function as a biochemical signal for regulating the stemness and differentiation of murine MuSCs and myoblasts. Native Col6, but not its pepsin-resistant fragment, counteracts the early differentiation of myogenic cells by reducing the expression of differentiation marker genes and preserving stemness features, with inhibition of the canonical Wnt pathway. Our data indicate that extracellular Col6 acts as a soluble ligand in delaying early myogenic differentiation by regulating intracellular signals involved in adult myogenesis.

## Teaser

**Summary:** Collagen VI, a pivotal extracellular regulator of muscle homeostasis, counteracts myogenic differentiation and sustains stemness by modulating intracellular signals involved in myogenesis.

## INTRODUCTION

Satellite cells are the major pool of muscle stem cells (MuSCs) involved in the growth and repair of skeletal muscle. Following muscle injury, MuSCs exit from quiescence and proliferate, providing new myoblasts, which fuse each other and with pre-existing myofibers to regenerate the tissue (Bentzinger et al., 2013a). In the quiescent state, satellite cells reside in a specialized niche beneath the basal lamina and in close contact with the myofiber sarcolemma (Yin et al., 2013). The MuSC niche is endowed with finely regulated biochemical and biomechanical properties that guarantee the maintenance of MuSC quiescence and, at the same time, guide the different steps of MuSC proliferation, activation and differentiation (Kuang et al., 2008). One major component of the MuSC niche is the extracellular matrix (ECM), which significantly influences the environmental signals controlling MuSC function (Gattazzo et al., 2014; Loreti and Sacco, 2022). Several ECM proteins have been found to influence MuSC function, including collagen VI (Col6), collagen V, fibronectin, laminin-211, laminin-111, fibrillin 1 and tenascin C, thus providing valuable information for dissecting the roles of the ECM in muscle physiopathology (Bentzinger et al., 2013b; Urciuolo et al., 2013; Tierney et al., 2016; Baghdadi et al., 2018; Rayagiri et al., 2018).

Col6 is a large protein with a broad distribution in the ECM of different organs, including skin, muscles, tendons, blood vessels, joints and nerves (Cescon et al., 2015). Among ECM components, Col6 has a distinctive process of intracellular assembly made of several different steps. The process starts with the association of three genetically distinct polypeptide chains – α1(VI), α2(VI) and α3(VI) (encoded by *COL6A1*, *COL6A2* and *COL6A3*, respectively) – which form a triple-helical monomer (∼500 kDa) containing large N- and C-terminal globular domains and a relatively short collagenous stalk. These monomers then assemble into antiparallel dimers (∼1000 kDa) and tetramers (∼2000 kDa), which are finally secreted into the extracellular space, where they form a branched network of ‘beaded microfilaments’ (Colombatti et al., 1987; Colombatti et al., 1995; Baldock et al., 2003). The key role of Col6 in skeletal muscle is underlined by the fact that its deficiency leads to muscle pathology in both humans and mice (Jöbsis et al., 1996; Irwin et al., 2003; Grumati et al., 2010). Pathogenic variants of the *COL6A1–COL6A3* genes are causative for Col6-related myopathies, which are inherited muscle disorders primarily characterized by muscle weakness and wasting, joint hyperlaxity and multiple contractures, and for which a cure is not yet available (Bönnemann, 2011).

Within the MuSC niche, Col6 is expressed by quiescent satellite cells and by stromal cells, such as interstitial fibroblasts and fibro-adipogenic precursors, which are known to contribute to niche formation and remodeling (Zou et al., 2008; Urciuolo et al., 2013; Petrany et al., 2020). The contribution of Col6 to MuSC homeostasis and muscle regeneration has been highlighted by work in Col6-null mice, in which MuSCs are constitutively more activated and display lower self-renewal capability, linking these defects to altered biomechanical properties of the niche elicited by Col6 deficiency (Urciuolo et al., 2013). Yet, it is conceivable that Col6 may also convey important biochemical signals, given its ability to bind to several surface receptors (Cescon et al., 2015; Tonelotto et al., 2021). Here, we investigated the effects exerted directly by Col6 on the *in vitro* differentiation of both C2C12 myogenic cells and primary MuSCs derived from wild-type mice. Our results show that Col6 delays the early differentiation of myogenic cells by maintaining them in a more stem-like condition via inhibition of the Wnt signaling pathway.

## RESULTS AND DISCUSSION

### Col6 inhibits early *in vitro* differentiation of C2C12 myoblasts

**Fig. 1.**
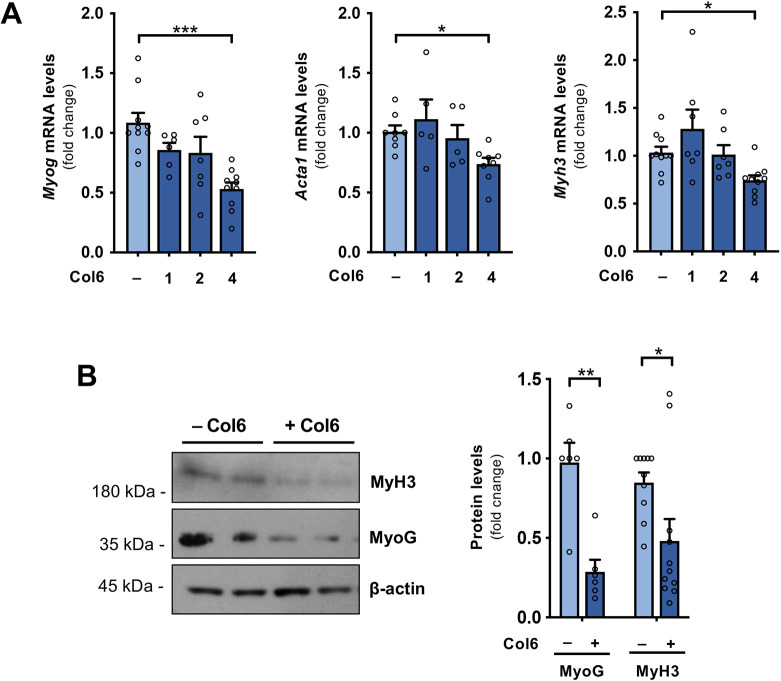
**Col6 inhibits *in vitro* differentiation of C2C12 myoblasts.** (A) RT-qPCR analysis of *Myog, Acta1* and *Myh3* mRNA levels in C2C12 cells maintained for 48 h in differentiating conditions (2% horse serum) in the absence of any treatment (–) or treated with different concentrations of soluble full-length Col6 (1, 2 and 4 µg/ml). mRNA levels are shown as fold change compared to the untreated condition (*n*=5–10 for each condition). (B) Representative western blot for MyH3 and MyoG protein levels in differentiating C2C12 cells maintained for 48 h in the absence (–) or in the presence (+) of 4 µg/ml Col6. β-actin was used as loading control. The two lanes for each condition represent different samples from separate cultures. The graph on the right shows the relative densitometric quantifications, as obtained from three independent western blot experiments (*n*=6–10 for each condition). Quantitative data are presented as mean+s.e.m. *P<0.05; **P<0.01; ***P<0.001 [one-way ANOVA test with Holm–Šidák post hoc test for multiple comparison (A); unpaired two-tailed Mann–Whitney test (B)].

We first used the C2C12 myogenic cell line to assess the capability of Col6 to modulate *in vitro* myogenic differentiation by culturing C2C12 cells in low-serum conditions for 48 h, when expression of differentiation markers is markedly increased (Fig. S1A), and evaluating the effects elicited by increasing amounts of purified Col6 directly added to the culture medium. Many commercially available collagen molecules are based on pepsin digestion of animal tissues; this procedure preserves triple-helical regions but is detrimental for the integrity of the whole protein and respective interactors, especially for those collagens with extended non-triple-helical regions, such as Col6. Therefore, we purified murine native Col6 using an ad hoc protocol and applied it as soluble protein for *in vitro* treatments, in comparison with the pepsin-resistant fragment of Col6. After 48 h of treatment, we detected significantly decreased expression of *Myog, Acta1* and *Myh3* differentiation marker genes in cultures treated with full-length Col6 (Fig. 1A), but not in those treated with the pepsin-resistant fragment of Col6, as compared to untreated controls (Fig. S1C). The Col6-mediated downregulation of myogenic differentiation genes was still maintained when cells were treated in the presence of inhibitory antibodies against β1 integrin (ITGB1), a known Col6 receptor that binds to the triple-helical region (Pfaff et al., 1993), thus suggesting that such interaction is not required for the transduction of these effects (Fig. S1D). Cell attachment assays showed significantly delayed cell adhesion to fibronectin, a major extracellular ligand for β1 integrin, when C2C12 cells were treated with the same inhibitory antibody, confirming the inhibition of β1 integrin binding (Fig. S1E).

Col6 also elicited significantly increased expression of *Pax7* (Fig. S1D), which encodes a key transcription factor for MuSC stemness (von Maltzahn et al., 2013). To delve deeper into the potential cell receptor(s) involved in the transduction of Col6 signals modulating myogenic differentiation, we used chemical inhibitors targeting different receptor classes for ECM ligands, including known cell surface interactors of Col6, such as RGD-dependent and RGD-independent integrins, and the discoidin domain receptors DDR1 and DDR2. Interestingly, none of the applied inhibitors was effective in reducing the inhibitory effects produced by Col6 (Fig. S2A–C), thus implying that integrins and discoidin domain receptors do not play substantial roles in mediating the differentiation inhibitory signals elicited by Col6 in C2C12 cells. The above findings were further supported by western blotting and immunofluorescence experiments, which showed a marked decrease in MyoG and MyH3 protein levels, and in the percentage of MyH3-positive cells in C2C12 cultures treated with full-length Col6 (Fig. 1B; Figs S1B and S2B,C).

Taken together, these results indicate that Col6 supplementation is able to counteract myoblast early differentiation, pointing to a role for Col6 in myogenesis. Therefore, we carried out further work aimed at establishing how Col6 regulates *in vitro* myogenesis, shifting our attention to primary MuSCs and the effects exerted by Col6 in the initial steps of MuSC differentiation.

### Col6 delays *in vitro* MuSC differentiation by maintaining stemness properties

**Fig. 2.**
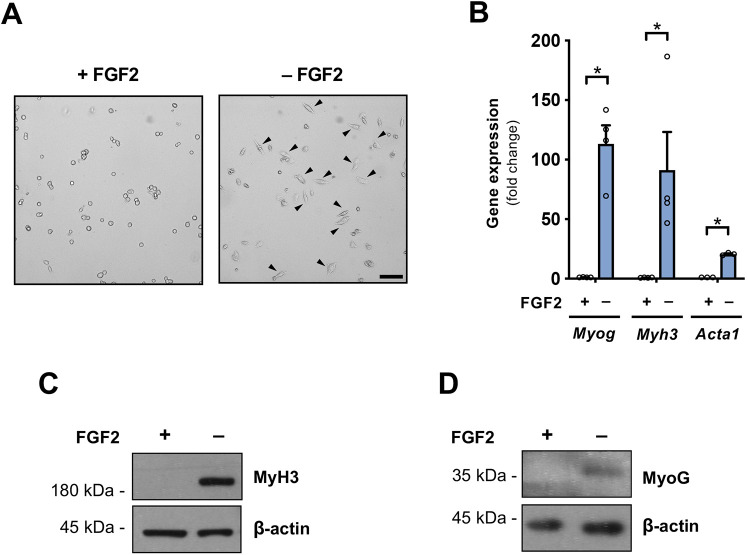
**Characterization of satellite cell-derived cultures of primary mouse MuSCs.** (A) Representative bright-field images of MuSCs isolated from wild-type mice and cultured for 48 h in the presence (+) or absence (–) of 25 ng/ml FGF2. Arrowheads point to differentiating cells with elongated, myoblast-like shape. Scale bar: 100 µm. Images are representative of three independent experiments. (B) Gene expression analysis for myogenic differentiation markers, as determined by RT-qPCR of primary MuSCs cultured for 48 h in the presence (+) or absence (–) of 25 ng/ml FGF2. mRNA levels are shown as fold change compared to the respective +FGF2 condition (*n*=3–4 for each condition). Data are presented as mean+s.e.m. *P<0.05 (unpaired two-tailed Mann–Whitney test). (C,D) Representative western blots for (C) MyH3 and (D) MyoG in protein extracts of primary MuSCs cultured for 48 h in the presence (+) or absence (–) of 25 ng/ml FGF2. β-actin was used as loading control. Blots are representative of three independent experiments.

To explore the effects exerted by Col6 on MuSC differentiation, we first implemented an optimal *in vitro* experimental setup for such studies. Indeed, it is well known that when isolated from muscles and cultured *in vitro*, MuSCs lose their quiescent state and enter a proliferative phase; therefore, secreted factors regulating MuSC behavior in their niche, such as fibroblast growth factor 2 (FGF2), insulin-like growth factors, hepatocyte growth factor and activators of Notch signaling, are routinely used to sustain MuSC stemness and prevent spontaneous *in vitro* differentiation (Yin et al., 2013). We isolated satellite cells from wild-type mice and used them to establish primary MuSC cultures. Withdrawal of FGF2 for 48 h was sufficient to induce modification of cell shape, which became elongated and myoblast-like (Fig. 2A). FGF2 withdrawal also led to increased expression of myogenic differentiation genes, as well as to strong downregulation of tenascin C (*Tnc*) gene expression, which is known to promote MuSC stemness and self-renewal (Tierney et al., 2016) (Fig. 2B; Fig. S3A), thus confirming that differentiation was prompted. This was paralleled by a drop in proliferation rates, as illustrated by the decreased number of Ki-67-positive nuclei (Fig. S3B) and by the increased MyH3 and MyoG protein levels following FGF2 withdrawal (Fig. 2C,D).

**Fig. 3.**
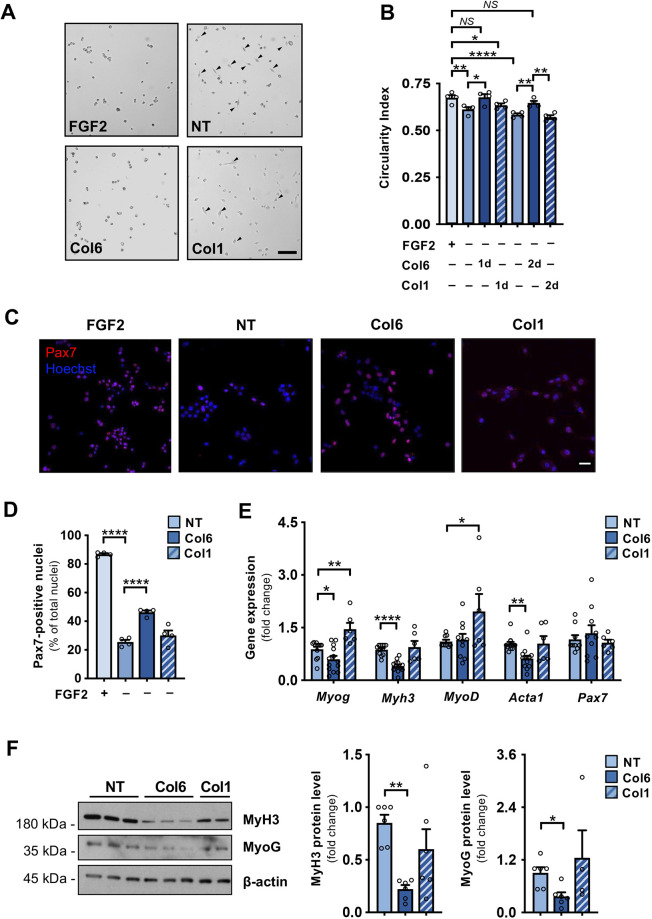
**Col6 delays *in vitro* MuSC differentiation and sustains stemness.** (A) Representative bright-field images of primary differentiating MuSCs cultured for 48 h either with 25 ng/ml FGF2 or under FGF2 withdrawal conditions in the absence (NT) or presence of 4 µg/ml Col6 or 4 µg/ml Col1, as indicated. Arrowheads point to differentiating cells with elongated, myoblast-like shape. Scale bar: 100 µm. (B) Morphometric analysis of primary MuSCs cultured for 48 h in the presence of 25 ng/ml FGF2 (FGF2+), or maintained under FGF2 withdrawal conditions (FGF−) with or without 4 µg/ml Col6 or 4 µg/ml Col1 for 24 h (1d, columns 2–4) or 48 h (2d, columns 5–7), as indicated. The circularity indexes range from 0 (highly elongated shape) to 1 (fully circular shape). At least 500 cells were analyzed for each condition (*n*=4 for each condition). (C) Representative immunofluorescence for Pax7 (red) in primary MuSCs maintained for 48 h in the presence of 25 ng/ml FGF2 (FGF2), or in the absence of FGF2 without any treatment (NT), or in the absence of FGF2 and treated with either 4 µg/ml Col6 or 4 µg/ml Col1. Nuclei were counterstained with Hoechst 33258 (blue). Scale bar: 50 µm. (D) Quantification of Pax7-positive nuclei as a percentage of total nuclei in primary MuSCs cultured for 48 h in the presence (+) or absence (–) of 25 ng/ml FGF2, and without any treatment (NT) or treated with 4 µg/ml Col6 or 4 µg/ml Col1, as indicated. From 300 to 1300 cells were analyzed for each condition (*n*=4 for each condition). (E) Gene expression analysis for myogenic differentiation (*Myog*, *Myh3*, *Myod*, *Acta1*) and stemness (*Pax7*) markers, as determined by RT-qPCR of primary MuSCs maintained for 48 h in the absence of FGF2 and either without any treatment (NT) or treated with 4 µg/ml Col6 or 4 µg/ml Col1, as indicated. mRNA levels are shown as fold change compared to the respective untreated condition (*n*=6–12 for each condition). (F) Representative western blot for MyH3 and MyoG in protein extracts of primary MuSCs maintained for 48 h in the absence of FGF2 and without any treatment (NT) or treated with either 4 µg/ml Col6 or 4 µg/ml Col1. β-actin was used as loading control. The multiple lanes for each condition represent different samples from separate cultures. Graphs on the right show the relative densitometric quantifications, as obtained from three independent western blot experiments (*n*=4–6 for each condition). Quantitative data are presented as mean+s.e.m.*P<0.05; **P<0.01*;* ****P<0.0001; NS, not significant (one-way ANOVA test with Holm–Šidák post hoc test for multiple comparison used in B, D, E, F).

Based on these data, we used the same primary MuSC cultures to investigate the effects elicited by supplementation of Col6, or of collagen I (Col1) used as a control, upon FGF2 withdrawal for 24 or 48 h. Morphometric analysis of primary MuSC cultures under differentiating conditions revealed that Col6 administration inhibits cell elongation, as highlighted by a significant increase in the circularity index of Col6-treated cultures when compared to the corresponding untreated cultures (Fig. 3A,B). Notably, such increase was displayed upon both 24 h and 48 h treatments with full-length Col6, and the circularity index was not significantly different from that of control cultures maintained in the presence of FGF2 (Fig. 3B). In agreement with this, the percentage of Pax7-positive cells, which as expected was high in the presence of FGF2 and dramatically decreased upon FG2 withdrawal, was partially restored by Col6 treatment but not by Col1 treatment (Fig. 3C,D), thus highlighting the capability of Col6 to maintain a higher stemness rate even in the absence of FGF2. This conclusion was also supported by the expression of myogenic markers, as we observed significantly decreased levels of *Myh3*, *Myog* and *Acta1* transcripts (Fig. 3E), and of MyH3 and MyoG proteins (Fig. 3F) in Col6-treated cultures. Intriguingly, cultures treated with Col1 displayed an opposite effect, having significantly higher levels of *Myog* and *Myod* transcripts than untreated cultures (Fig. 3E). Furthermore, the stemness markers syndecan-3 and tenascin C (Pisconti et al., 2016; Tierney et al., 2016) showed significantly higher expression levels in Col6-treated cultures, as compared to levels in the corresponding untreated cultures (Fig. S3C).

This set of data shows that soluble Col6 counteracts early *in vitro* differentiation of MuSCs, maintaining them in a less differentiated state. These findings strengthen the concept that Col6 is a critical regulator of MuSC stemness, revealing that in addition to a fine regulation of the mechanical properties of the MuSC niche exerted through the branched network of Col6 microfilaments deposited in the ECM (Urciuolo et al., 2013), native Col6 is capable of delaying early myogenic differentiation in a stiffness-independent fashion.

### Col6 treatment negatively regulates canonical Wnt signaling

**Fig. 4.**
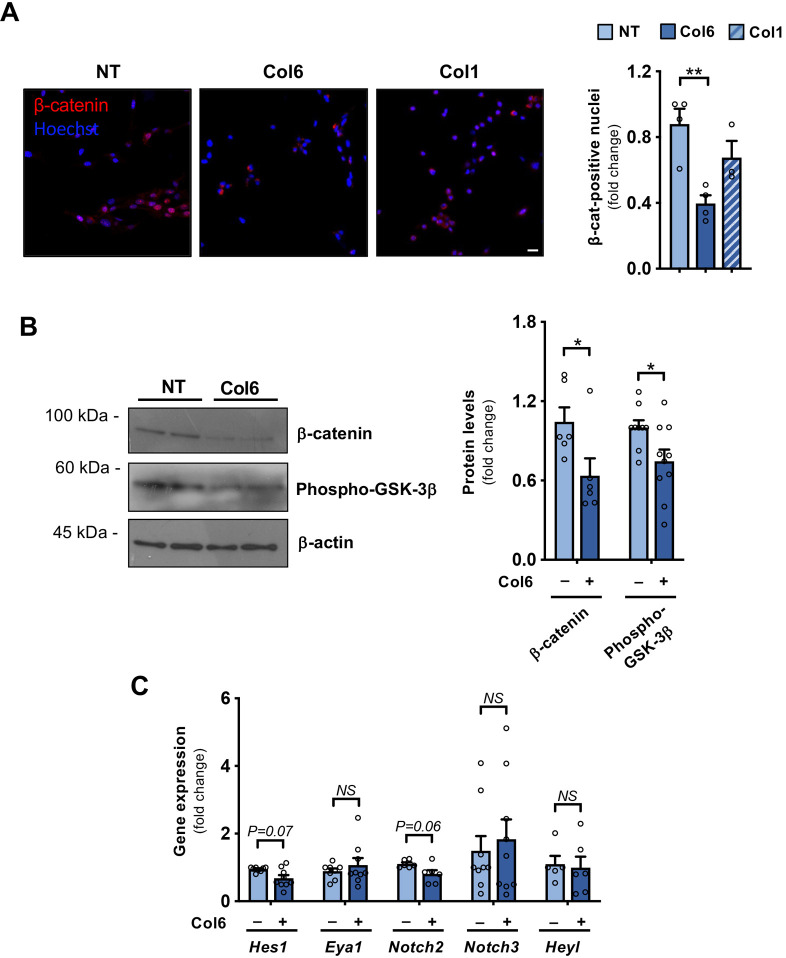
**Col6 treatment inhibits Wnt signaling without any significant induction of Notch signaling.** (A) Representative immunofluorescence for β-catenin (red) in primary MuSCs maintained for 48 h in the absence of FGF2 and without any treatment (NT) or treated with either 4 µg/ml Col6 or 4 µg/ml Col1. Nuclei were counterstained with Hoechst 33258 (blue). Scale bar: 50 µm. Graph on the right shows quantification of β-catenin-positive nuclei, shown as fold change compared to the untreated condition. From 115 to 215 cells were analyzed for each condition (*n*=3–4 for each condition). (B) Representative western blotting for β-catenin and phospho-GSK-3β protein levels in primary MuSCs maintained for 48 h in the absence of FGF2 without any treatment (NT) or treated with 4 µg/ml Col6. β-actin was used as loading control. The two lanes for each condition represent different samples from separate cultures. The graph on the right shows the relative densitometric quantifications, as obtained from three independent western blot experiments (*n*=6–9 for each condition). (C) Gene expression analysis of Notch downstream targets, as determined by RT-qPCR of primary MuSCs maintained for 48 h under FGF2 withdrawal in the absence (–) or presence (+) of 4 µg/ml Col6. mRNA levels are shown as fold change compared to the respective untreated condition (*n*=5–9 for each condition. Quantitative data are presented as mean+s.e.m. *P<0.05; **P<0.01; NS, not significant [one-way ANOVA test with Holm–Šidák post hoc test for multiple comparison (A); unpaired two-tailed Mann–Whitney test (B,C)].

The maintenance of MuSC stemness is strictly regulated by a balance between Notch and Wnt signaling pathways, in which the first favors dormancy (Bi et al., 2016; Baghdadi et al., 2018), while the second promotes myogenic differentiation (Brack et al., 2008; Brack et al., 2009). Therefore, we explored whether the Col6-dependent effects on *in vitro* MuSC differentiation rely on modulation of these pathways. We first assessed the subcellular localization of β-catenin (CTNNB1), the major transcriptional coactivator of the canonical Wnt pathway. Immunofluorescence and western blotting showed that treatment of differentiating MuSC cultures with full-length Col6 significantly decreases the amount of nuclear β-catenin (Fig. 4A) as well as β-catenin total protein levels (Fig. 4B). As expected, treatment with Wnt-3a, used as a positive control, led to a distinct nuclear localization of β-catenin in differentiating MuSC-derived cultures (Fig. S4A). We next assessed the activation state of glycogen synthase kinase 3 (GSK-3) kinases, which are negative regulators of Wnt signaling that target β-catenin for degradation, and found a significant decrease in the inhibitory phosphorylation of GSK-3β in Col6-treated cultures when compared to levels in the corresponding control cultures (Fig. 4B), pointing to downregulation of the Wnt pathway.

To corroborate these findings, we analyzed the expression of prospero-related homeobox1 (Prox1), a transcription factor that plays an essential role in MuSC differentiation and whose activity is strictly regulated by the Wnt–β-catenin pathway (Kivelä et al., 2016). Expression of the *Prox1* gene was strongly upregulated in MuSC cultures upon FGF2 withdrawal, in agreement with its role in promoting myogenic differentiation (Fig. S4B). Col6 treatment led to a significant decrease in *Prox1* expression in differentiating MuSCs (Fig. S4C), again supporting the concept that the effects elicited by Col6 supplementation involve modulation of the Wnt–β-catenin pathway. On the other hand, the Col6-dependent effects in enhancing stemness and delaying differentiation of primary MuSC cultures do not appear to primarily involve the Notch signaling pathway, at least in this experimental setup. Indeed, the expression levels of several major Notch target genes (*Notch2, Notch3, Eya1, Hes1* and *Heyl*) were not significantly different between Col6-treated and untreated cultures maintained under the same differentiating conditions as above (Fig. 4C), pointing to marginal or absent involvement of this pathway in the effects elicited by Col6. Taken together, the above set of data reveal that Col6 treatment of differentiating MuSC cultures inhibits the Wnt–β-catenin pathway, leading in turn to decreased differentiation and higher maintenance of the stemness state.

In conclusion, these results provide new information on the mechanisms involved in stem cell–niche interactions, offering some novel concepts about the diverse roles of Col6 in skeletal muscle, as well as for muscle homeostasis and regeneration. Future work aimed at the use of native Col6 and its various domains with different *in vitro* and *ex vivo* models and experimental setups will shed light on the Col6 subregions involved in the effects exerted by this key ECM molecule in the different muscle cell types, and will also assist in the identification of surface receptors and ligands involved in the transduction of outside–in signals triggered by Col6 in myofibers, satellite cells and stromal cells, which is an essential step for the development of targeted therapeutic strategies in Col6-related myopathies and other muscle disorders.

## MATERIALS AND METHODS

### Satellite cell isolation and primary MuSC cultures

Satellite cells were isolated from the extensor digitorum longus muscle of 1- to 2-month-old wild-type mice of the C57/BL6N strain. Muscles were cleared from tendons and fat tissue, and then chopped with scissors and a scalpel. The obtained specimens were chemically digested twice with 15 ml phosphate-buffered saline (PBS) supplemented with collagenase I (100 U/ml; Worthington, Lakewood, NJ, USA) and dispase (2 U/ml; Thermo Fisher Scientific, Waltham, MA, USA) for 20 min at 37°C with agitation. The solution was filtered through a 70 μm cell strainer to eliminate residues of muscle debris and then centrifuged at 1200 ***g*** for 30 min. The cell pellet was resuspended in Dulbecco's Modified Eagle's Medium (DMEM; Thermo Fisher Scientific) supplemented with 20% fetal bovine serum (FBS; Thermo Fisher Scientific) and plated onto 0.1% gelatin-precoated dishes (Sigma-Aldrich, St Louis, MO, USA) for 1 h at 37°C in 5% CO_2_, to deplete muscle fibroblasts and other cells. Following this pre-plating step, cells remaining in suspension were plated onto 0.1% gelatin-precoated dishes and cultured in DMEM medium supplemented with 20% FBS, 1% glutamine (Thermo Fisher Scientific), 1% penicillin-streptomycin (Thermo Fisher Scientific) and 5% chicken embryo extract (Life Science Production, Bedford, UK) at 37°C in 5% CO_2._ After the first passage, MuSCs were cultured in Ham's F-10 medium (Thermo Fisher Scientific) supplemented with 10% FBS, 1% glutamine, 1% penicillin-streptomycin and 25 ng/ml recombinant FGF2 (Wellcome-MRC Cambridge Stem Cell Institute, Cambridge, UK) to maintain them in a stem-like and proliferating condition. Differentiation was induced by FGF2 withdrawal and culture in the same medium as above. Only the second to fourth passages from initial isolation were used for all the experiments. Primary MuSCs preparations that were minorly contaminated by muscle fibroblasts were discarded and not used. All animal experiments were performed according to approved guidelines. Mouse procedures were approved by the Animal Ethics Committee of the University of Padova and by the Italian Ministry of Health (n. 100/2020-PR).

### C2C12 cells

C2C12 (CRL-1772; ATCC, Manassas, VA, USA) cells were used as an immortalized murine myogenic cell line, and were cultured in DMEM medium supplemented with 20% FBS, 1% glutamine and 1% penicillin-streptomycin at 37°C in 5% CO_2_. To induce myoblast differentiation and fusion, C2C12 cells were cultured for 2 days in DMEM medium supplemented with 2% horse serum (Merck), 1% glutamine and 1% penicillin-streptomycin at 37°C in 5% CO_2_. Both C2C12 and primary MuSC cultures were routinely tested for mycoplasma contamination and visually inspected for morphology and signs of bacterial contamination.

### Cell treatments

The triple-helical pepsin-resistant fragment of Col6 was purified from mouse tissues by pepsin digestion, fractionated salt precipitation and molecular sieve chromatography, as previously described (Trüeb et al., 1987; Bonaldo et al., 1998). Native full-length Col6 (whole tetrameric molecule) was purified from new-born wild-type mice by fractionated salt precipitation and molecular sieve chromatography, following established procedures and as previously described (Colombatti et al., 1989; Sabatelli et al., 2001). Both preparations were tested for purity by SDS-PAGE. Col1 was purchased from Sigma-Aldrich. Cells were treated with either full-length Col6, pepsin-resistant Col6 fragment or Col1, added as soluble molecules to the culture medium. Inhibitory antibodies against β1 integrin (rabbit monoclonal, 1798-1; Epitomics, Burlingame, CA, USA) were used at 10 μg/ml in culture medium. For cell attachment assays, C2C12 cells were preincubated with 10 μg/ml β1 integrin inhibitory antibody for 30 min in culture medium before plating onto dishes coated with fibronectin (5 μg/cm^2^; F1056, Sigma-Aldrich). Images were taken at 5, 10, 15, 30 and 90 min after plating. For treatments with chemical inhibitors, C2C12 cells were maintained for 2 days in culture medium supplemented with the following compounds: TC-I15 (100 μM; Tocris Bioscience, Bristol, UK), RGDS peptide (100 μM; Tocris Bioscience), RGES peptide (100 μM; Abbexa, Cambridge, UK), 7rh (20 nM; Tocris Bioscience) and WRG-28 (300 nM; Tocris Bioscience). Where indicated, MuSCs were treated with 10 ng/ml recombinant murine Wnt-3a (Peprotech, Cranbury, NJ, USA) for 48 h in differentiation medium.

### Quantitative real-time RT-PCR

For quantitative real-time RT-PCR (RT-qPCR), total RNA was extracted from cell samples using 0.5 ml TRIzol reagent (Thermo Fisher Scientific) and quantified using a Nanodrop ND-1000 (NanoDrop Technologies, Wilmington, DE, USA). Reverse transcription was performed with 500 ng total RNA and M-MLV Reverse Transcriptase (Thermo Fisher Scientific), using random hexamers, as previously described (Da Ros et al., 2022). The resulting cDNAs were processed for quantitative real-time PCR using Rotor-Gene SYBR Green PCR Kit mastermix (Qiagen, Hilden, Germany) and a Rotor-GeneQ thermocycler (Qiagen). Each sample was loaded in triplicate and analyzed with RotorGene Q 2.0.24 software. *Rps16* (*S16)*, coding for ribosomal protein S16, was used as a housekeeping gene control. Primer sequences are provided in Table S1.

### Western blotting

C2C12 and primary MuSCs were lysed in RIPA buffer (20 mM Tris-HCl, pH 7.5, 150 mM NaCl, 1 mM EGTA, 1% NP-40, 1% sodium deoxycholate) supplemented with protease and phosphatase inhibitors (Sigma-Aldrich) and stored at −80°C until use. Protein concentration was determined by the BCA Protein Assay Kit (Thermo Fisher Scientific), and 30–40 μg of each protein sample was subjected to SDS-PAGE in polyacrylamide Novex NuPAGE Bis-Tris 4–12% gels (Thermo Fisher Scientific), followed by electrotransfer onto polyvinylidene difluoride membranes (Millipore), as previously described (Metti et al., 2020). Membranes were stained with Ponceau (Sigma-Aldrich) and blocked for 1 h with 5% non-fat dry milk (Bio-Rad, Hercules, CA, USA) in Tris-buffered saline solution containing 0.1% Tween 20 (TBS-T), and then incubated overnight at 4°C with the primary antibodies, as previously described (Gambarotto et al., 2022). The following primary antibodies were used: mouse monoclonal anti-MyH3 (1:500; F1.652; Developmental Studies Hybridoma Bank, Iowa City, IA, USA), mouse monoclonal anti-MyoG (1:300; sc-12732; Santa Cruz Biotechnology, Dallas, TX, USA), rabbit polyclonal anti-phospho-GSK-3α/β (Ser 21/9) (1:1000; 9331S; Cell Signaling Technology, Danvers, MA, USA), rabbit monoclonal anti-β-catenin (1:500; ab32572; Abcam, Cambridge, UK), and mouse monoclonal anti-β-actin (1:3000; A5316; Sigma-Aldrich). After three washes in TBS-T, membranes were incubated for 1 h with horseradish peroxidase-conjugated anti-rabbit IgG or anti-mouse IgG secondary antibodies (1:1000; Bethyl Laboratories, Montgomery, TX, USA). Detection was carried out by using SuperSignal West Pico (Thermo Fisher Scientific). Densitometric quantification was performed using Fiji software (Rueden et al., 2017). Uncropped gels used in the figures are displayed in supplementary material for blot transparency (Fig. S5).

### Immunofluorescence

For immunofluorescence analyses, C2C12 cells were plated in 24-well plates on glass coverslips, whereas primary MuSCs were cultivated in 8-well chamber slides (Thermo Fisher Scientific). Cells were fixed with 4% paraformaldehyde at room temperature for 2 min and washed three times in PBS for 5 min. Permeabilization was performed using cold (−20°C) methanol for 5 min for C2C12 cells or 0.3% Triton X-100 in PBS for 10 min for primary MuSCs. Slides were washed three times in PBS and then incubated for 1 h with 10% goat serum (Merck) in PBS, to block unspecific binding of antibodies. Primary antibodies against Pax7 (1:25; mouse monoclonal; Pax7-c; Developmental Studies Hybridoma Bank), MyoG (1:200; rabbit monoclonal; ab124800; Abcam), MyH3 (1:200; mouse monoclonal; F1.652; Developmental Studies Hybridoma Bank), Ki-67 (1:100; rabbit monoclonal; NB110-89717; Novus Biologicals, Centennial, CO, USA) and β-catenin (1:200; rabbit polyclonal; ab32572; Abcam) were diluted in 5% goat serum in PBS and incubated at 4°C overnight in a humidified chamber. Slides were washed three times in PBS and incubated for 1 h with anti-rabbit IgG Cy2- or Cy3-conjugated (1:500; Jackson ImmunoResearch, West Grove, PA, USA) and anti-mouse IgG Cy3-conjugated (1:500; Jackson ImmunoResearch) secondary antibodies. Nuclei were counterstained with Hoechst 33258 (Sigma-Aldrich). After three washes in PBS, slides were finally mounted in 80% glycerol in PBS.

### Morphometric analysis

Bright-field micrographs were captured with a Leica DMI4000 inverted microscope at 10× objective magnification, and the circularity index was calculated using FIJI software by setting a range extending from 0 (highly elongated shape) to 1 (circular shape) (Rueden et al., 2017; Eliazer et al., 2019).

### Statistics

All results are shown as mean+s.e.m. Statistical analysis of data was performed by unpaired two-tailed Mann–Whitney test (GraphPad). For experiments with more than two conditions, one-way or two-way analysis of variance (ANOVA) was used (GraphPad). When ANOVA revealed significant differences, further analysis was carried out using Holm–Šídák multiple comparison tests. Statistical significance was set at *P*<0.05. All bar graphs display individual values, and the numbers of biological replicates (always greater than three) are indicated in the figure legends.

## Supplementary Material



10.1242/joces.261419_sup1 Supplementary information

## First Person

This article has an associated First Person interview with Samuele Metti, joint first author of the paper.
