# Supplementary material for: Native collagen VI delays early muscle stem cell differentiation
Source: J Cell Sci. 2024 Feb 12;137(3):jcs261419. doi: 10.1242/jcs.261419 (PMC10911284; doi:10.1242/jcs.261419)
Supplement: Supplementary information [file joces-137-261419-s1.pdf]

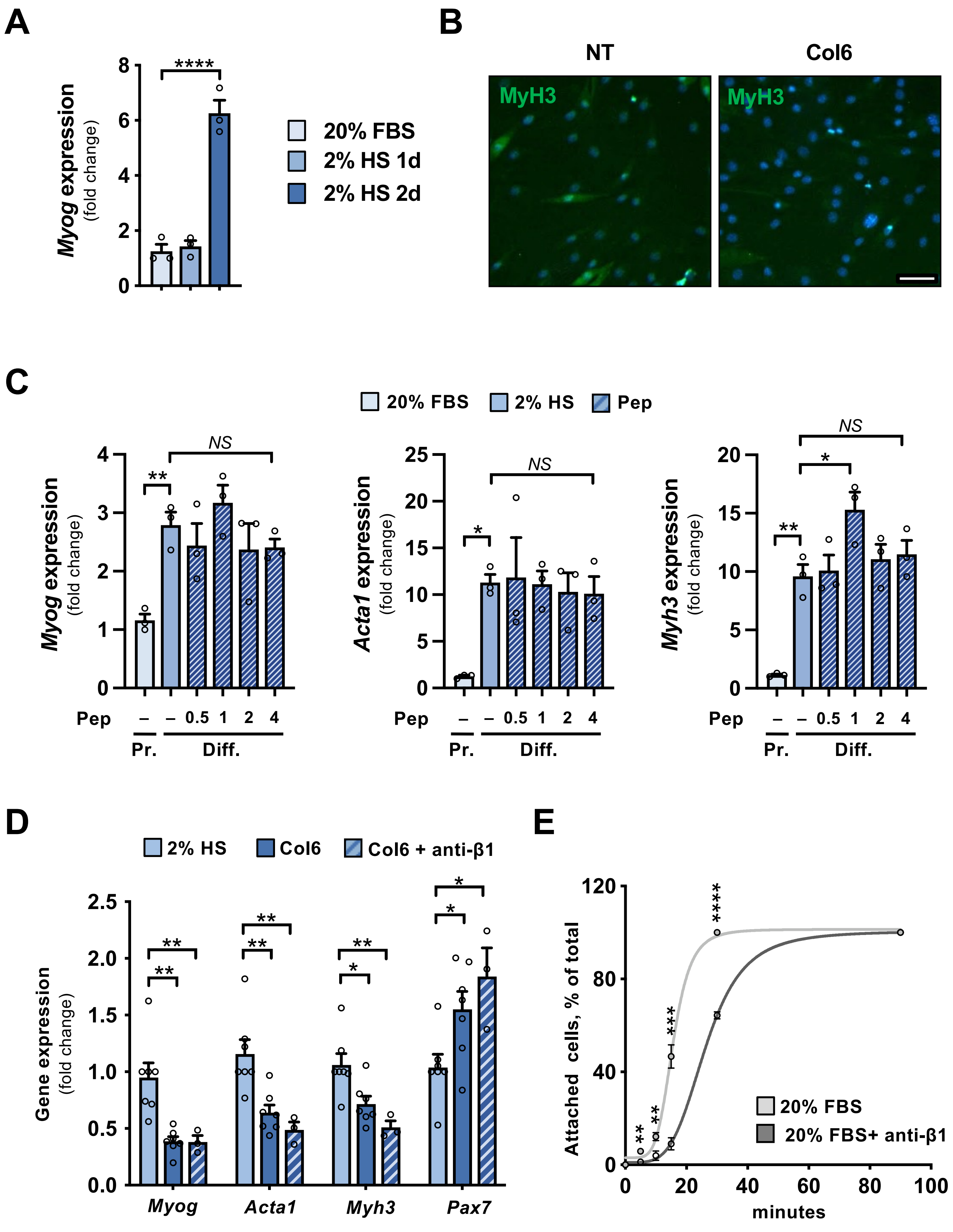

Journal of Cell Science • Supplementary information

Fig. S1. (**A**) RT-qPCR analysis of *Myog* mRNA levels in proliferating C2C12 cells (20% FBS) and in differentiating C2C12 cells at 1 day (2% HS 1d) and 2 days (2% HS 2d) of differentiation. mRNA levels are shown as fold change compared to the proliferating condition (*n*=3 for each condition). Data are presented as mean+s.e.m. ****P<0.0001 (one-way ANOVA test with Holm-Šidák *post hoc* test for multiple comparison). (**B**) Representative immunofluorescence for MyH3 (green) in differentiating C2C12 cells maintained for 48 hr in the absence (NT) or in the presence (Col6) of 4 µg/ml Col6. Nuclei were counterstained with Hoechst (blue). Scale bar: 50 µm. (**C**) RT-qPCR analysis of *Myog, Acta1* and *Myh3* mRNA levels in C2C12 cells maintained for 48 hr in proliferating (Pr.) or in differentiating (Diff.) conditions, in the absence of any treatment (–) or treated with different concentrations of pepsin-resistant Col6 fragment (Pep 0.5, 1, 2 and 4 µg/ml). mRNA levels are shown as fold change compared to the proliferating (20% FBS) condition (*n*=3 for each condition). Data are presented as mean+s.e.m. *P<0.05; **P<0.01; NS, not significant (one-way ANOVA test with Holm-Šidák *post hoc* test for multiple comparison). (**D**) Gene expression analysis for myogenic differentiation (*Myog*, *Acta1*, *Myh3*) and stemness (*Pax7*) markers, as determined by RT-qPCR of differentiating C2C12 cells maintained for 48 hr in the absence of any treatment (2% HS), or treated with Col6 at 4 µg/ml (Col6), or with 4 µg/ml Col6 in combination with 10 µg/ml β1 integrin inhibitory antibodies (Col6 + anti-β1). mRNA levels are shown as fold change compared to the respective untreated condition (*n*=3-7 for each condition). Data are presented as mean+s.e.m. *P<0.05; **P<0.01 (one-way ANOVA test with Holm-Šidák *post hoc* test for multiple comparison). (**E**) Percentage of attached C2C12 cells cultured for the indicated time points in proliferating medium in the absence (20% FBS) or presence (20% FBS + anti-β1) of β1 integrin inhibitory antibodies (*n*=3 for each condition). Data are presented as mean+s.e.m. **P<0.01; ***P<0.001; ****P<0.0001 (unpaired two-tailed Mann–Whitney test).

Journal of Cell Science • Supplementary information

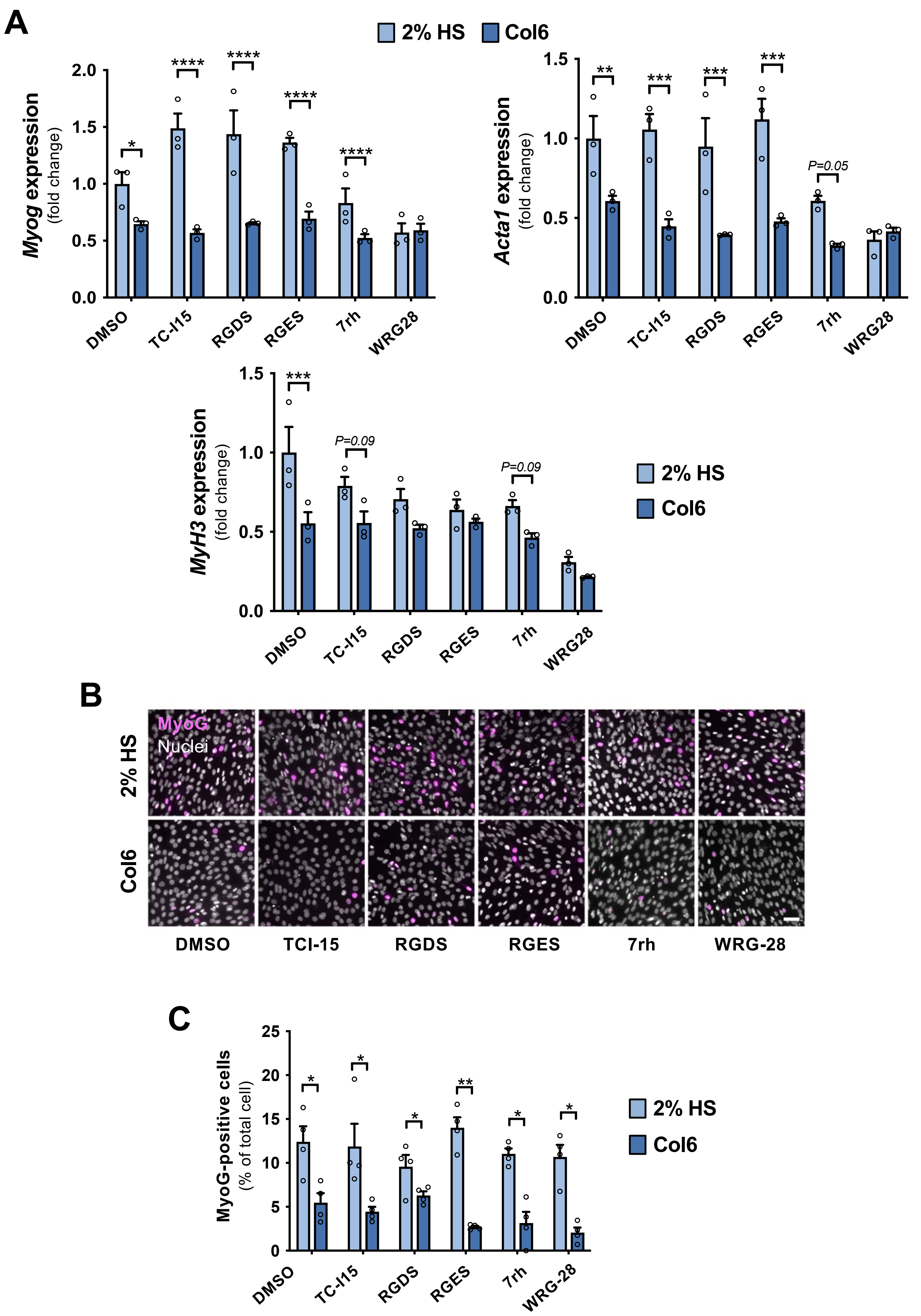

Journal of Cell Science • Supplementary information

Fig. S2. (**A**) RT-qPCR analysis of *Myog, Acta1* and *Myh3* mRNA levels in C2C12 cells maintained for 48 hr in differentiating conditions in the absence of any treatment (2% HS) or treated with Col6 at 4 µg/ml (Col6) in combination with the indicated membrane receptor inhibitors (TC-I 15 as an inhibitor of α2β1, α1β1 and α11β1 RGD-independent integrins; RGDS as a broad-spectrum antagonist of RGD-dependent integrins; 7rh and WRG-28 as selective inhibitors of DDR1 and DDR2 collagen receptors, respectively. RGES was as used a negative control for RGDS, whereas DMSO was used as a control for TC-I 15, 7rh and WRG-28. mRNA levels are shown as fold change compared to the DMSO condition (n=3 for each condition). Data are presented as mean+s.e.m. *P<0.05; **P<0.01; ***P<0.001; ****P<0.0001 (unpaired two-tailed Mann–Whitney test). (**B**) Representative immunofluorescence for MyoG (magenta) in C2C12 cells maintained for 48 hr in differentiating conditions in the absence of any treatment (2% HS) or treated with Col6 at 4 µg/ml (Col6) in combination with the above membrane receptors inhibitors and the respective controls. Nuclei were counterstained with Hoechst (white). Scale bar: 50 µm. (**C**) Quantification of the percentage of MyoG-positive cells in C2C12 cells maintained for 48 hr in differentiating conditions in the absence of any treatment (2% HS) or treated with Col6 at 4 µg/ml (Col6) in combination with the above membrane receptors inhibitors and the respective controls (n=3 for each condition). Data are presented as mean+s.e.m.

*P<0.05; **P<0.01 (unpaired two-tailed Mann–Whitney test).

Journal of Cell Science • Supplementary information

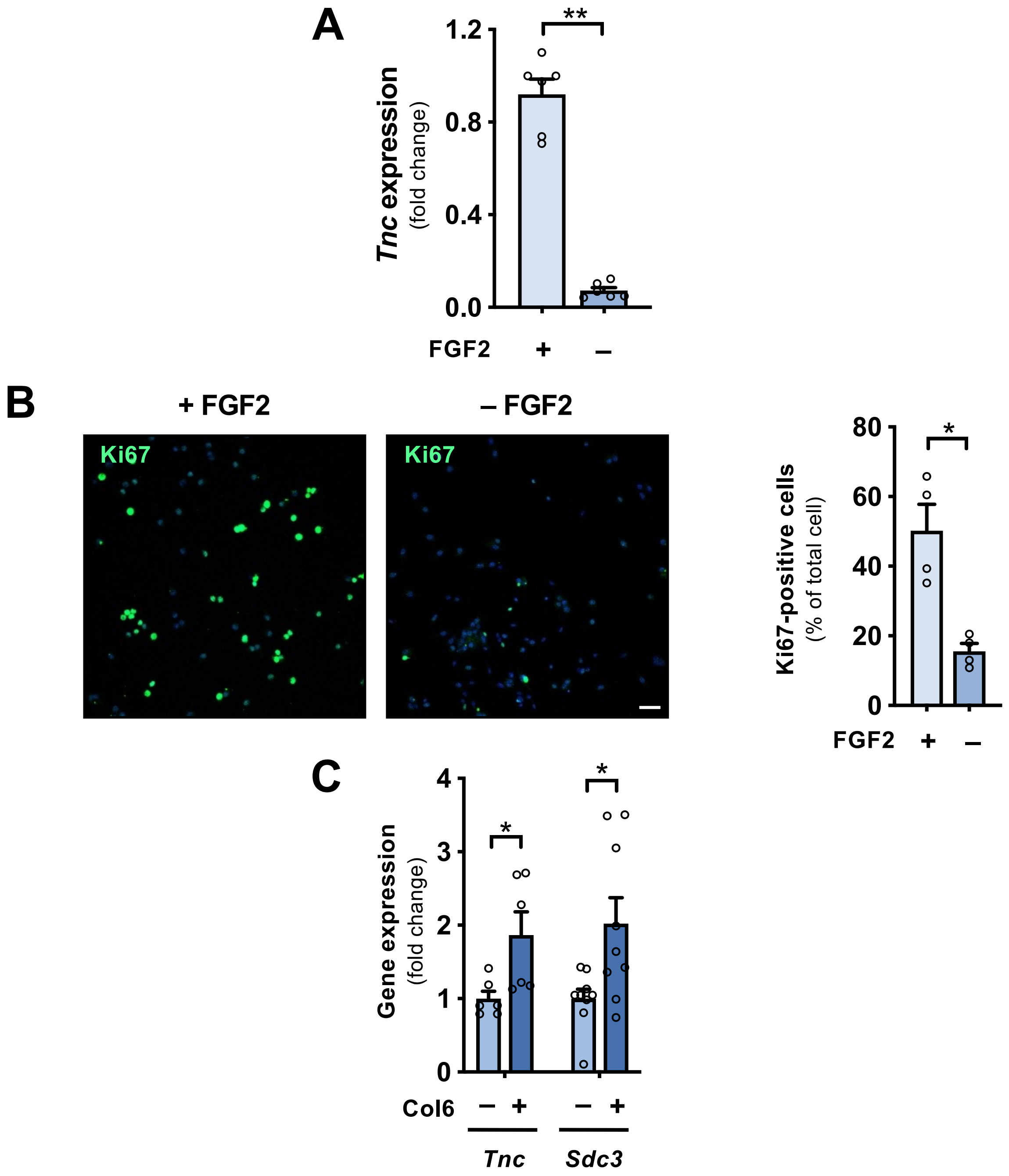

Fig. S3. (**A**) RT-qPCR analysis of *Tnc* mRNA levels in proliferating primary muscle stem cells (FGF2 +) and in differentiating primary muscle stem cells maintained for 48 hr in the absence of FGF2 (FGF2 –). mRNA levels are shown as fold change compared to the proliferating condition (*n*=6 for each condition). Data are presented as mean+s.e.m. ***P*<0.01 (unpaired two-tailed Mann–Whitney test). (**B**) Representative immunofluorescence for the proliferation marker Ki67 (green) in primary muscle stem cells cultured for 48 hr in the presence (+) or absence (–) of 25 ng/ml FGF2. Nuclei were counterstained with Hoechst (blue). Scale bar: 50 µm. The histogram on the right show the quantification of Ki67-positive cells on total cells (*n*=4 for each condition) *P<0.05. Data are presented as mean+s.e.m. (unpaired two-tailed Mann–Whitney test). (**C**) RT-qPCR analysis of *Tnc* and *Sdc3* mRNA levels in primary differentiating muscle stem cells cultured for 48 hr under FGF2 withdrawal and in the absence (–) or presence (+) of 4 µg/ml Col6. mRNA levels are shown as fold change compared to the respective untreated condition (*n*=6-9 for each condition). Data are presented as mean+s.e.m. *P<0.05 (unpaired two-tailed Mann–Whitney test).

Journal of Cell Science • Supplementary information

J. Cell Sci.: doi:10.1242/jcs.261419: Supplementary information

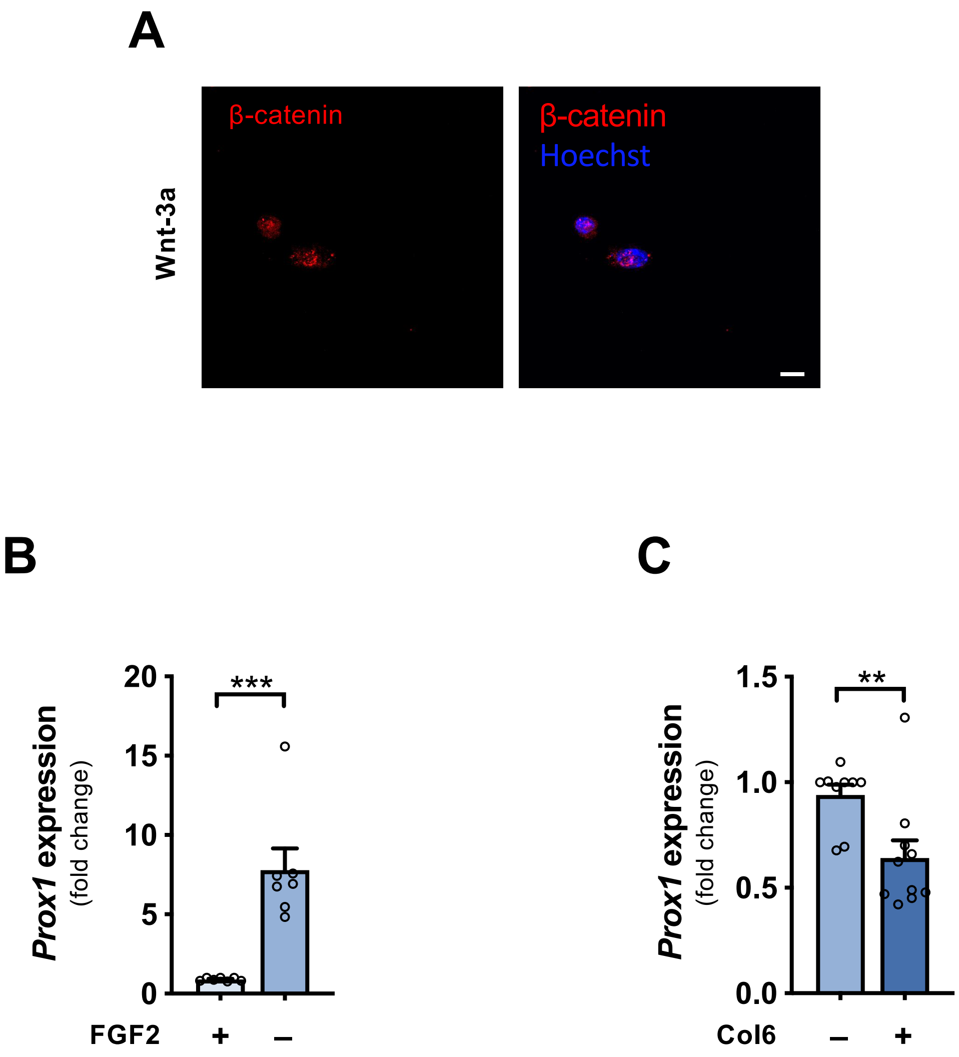

Fig. S4. (**A**) Representative immunofluorescence for β-catenin (red) in primary muscle stem cells maintained for 48 hr in the absence of FGF2 and treated with 10 ng/ml recombinant Wnt-3a. Nuclei were counterstained with Hoechst (blue). Scale bar: 10 µm. (**B**) RT-qPCR analysis of *Prox1* mRNA levels in proliferating primary muscle stem cells (FGF2 +) and in differentiating primary muscle stem cells maintained for 48 hr in the absence of FGF2 (FGF2 –). mRNA levels are shown as fold change compared to the proliferating condition (n=6-7 for each condition). Data are presented as mean+s.e.m. ***P<0.001 (unpaired two-tailed Mann–Whitney test). (**C**) RT-qPCR analysis of *Prox1* mRNA levels in primary muscle stem cells maintained for 48 hr under FGF2 withdrawal and in the absence (–) or presence (+) of 4 µg/ml Col6. mRNA levels are shown as fold change compared to the untreated condition (n=9-10 for each condition). Data are presented as mean+s.e.m. **P<0.01 (unpaired two-tailed Mann–Whitney test).

Uncropped gels for Fig. 1B

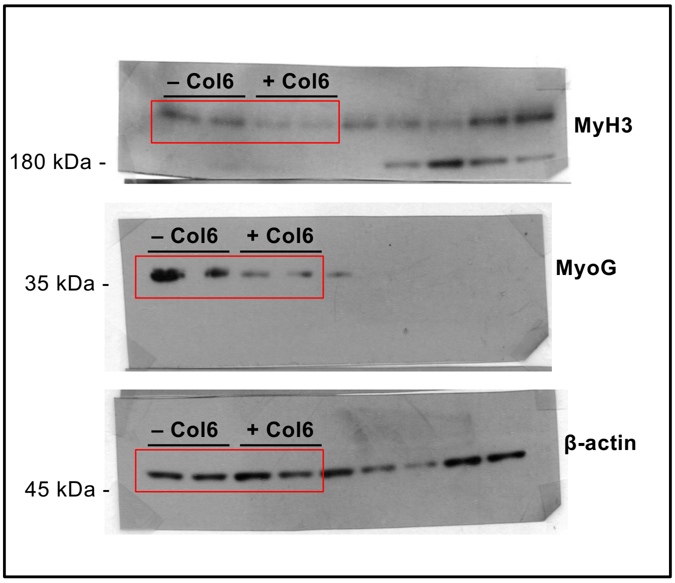

Uncropped gels for Fig. 2C

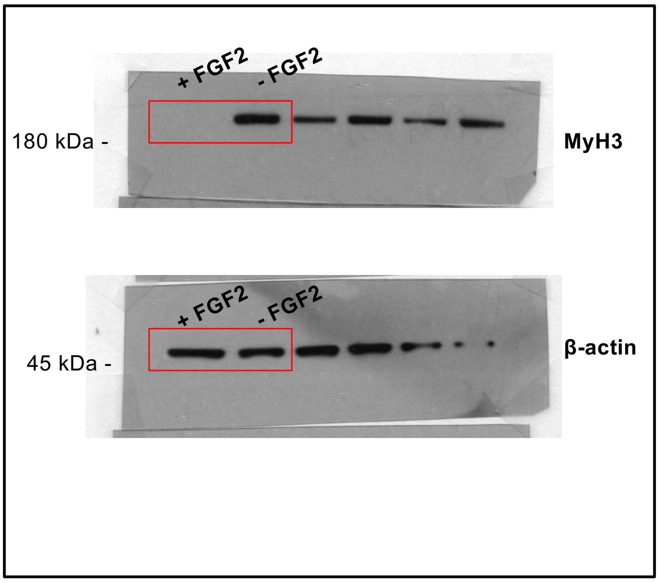

Uncropped gels for Fig. 2D

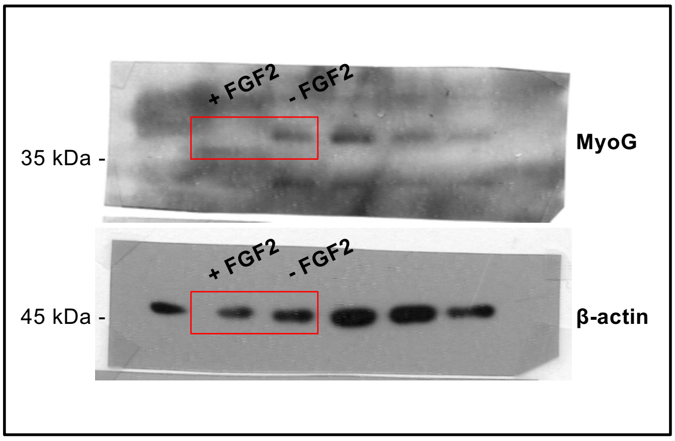

Uncropped gels for Fig. 3F

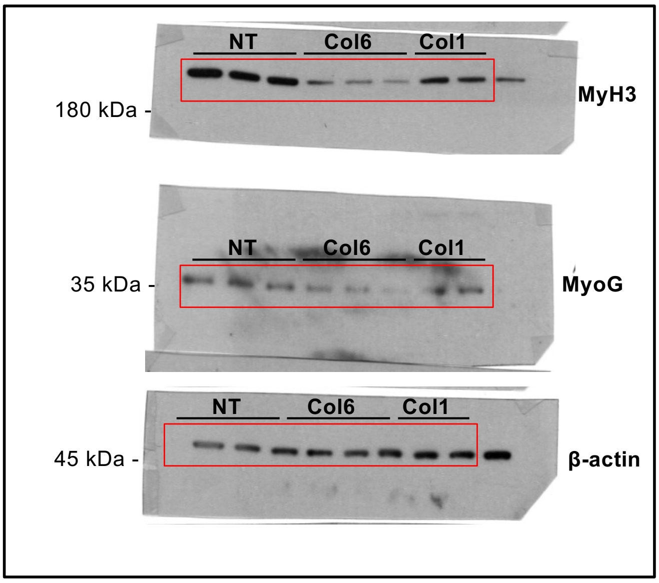

Uncropped gels for Fig. 4B

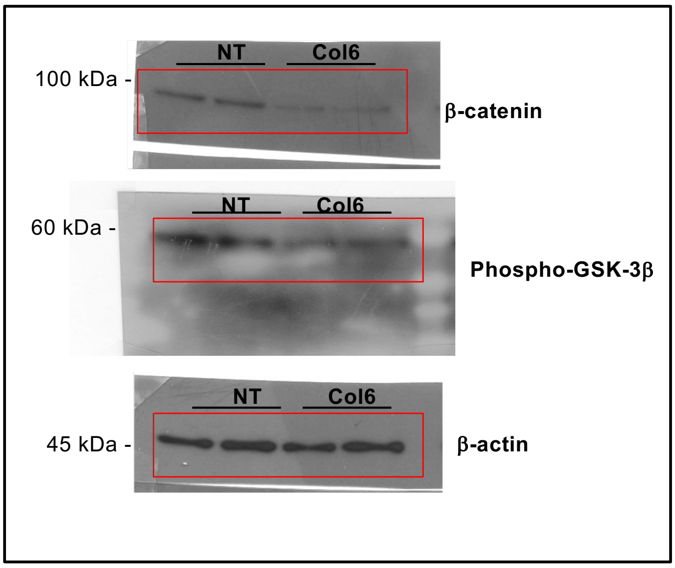

Fig. S5. Blot transparency.

Journal of Cell Science • Supplementary information

Table S1. List of primers used for RT-qPCR experiments.

	Forward (5' > 3')	Reverse (5' > 3')
Acta1	CCCACAACGTGCCCATCTAT	GATGTCGCGCACAATCTCAC
Eya1	CAGCAGACGGGTCTTTAGACA	GGTGAGCTGGTCTTGGACTAAA
Hes1	TGAAGCACCTCCGGAACC	CGCGGTATTTCCCCAACAC
Heyl	GTCCCCACTGCCTTTGAGAA	TCCACGGTCATCTGCAAGAC
Myh3	CGCAGAATCGCAAGTCAATA	ATATCTTCTGCCCTGCACCA
Myod	GCTGTCCCTGGTTCTTCACG	TCCTTTCTTTGGGGCTGGAT
Myog	CCCCACTCCCCATTCACATA	CTCCTGAGTTTGCCCCACTG
Notch2	TGGTTCTGGGACAAGTGAACA	ACAGCAAAGCCTCATCCTCA
Notch3	CCATGCCGATGTCAATGCA	TAGCCTCCACGTTGTTCACA
Pax7	CGCTGTGCTGGGACTTCTTC	AGACTCAGGGCTTGGGAAGG
Prox1	GCTACCCCAGCTCCAACATGCT	TGATGGCTTGACGCGCATACTTCT
Tnc	TGGGAAGACGCTAGGGACCG	GAAGAAGGATCTTTTCCAGGTCGG
Sdc3	CTCTGGCTACTTCGAGCAG	CTGGCTGGACTCTTCTACG
S16	GCAGTACAAGTTACTGGAGCC	CGGTAGGATTTCTGGTATCG

Journal of Cell Science • Supplementary information
