# Supplementary material for: Native collagen VI delays early muscle stem cell differentiation
Source: J Cell Sci. 2024 Feb 12;137(3):jcs261419. doi: 10.1242/jcs.261419 (PMC10911284; doi:10.1242/jcs.261419)
Supplement: Supplementary file 1 [file joces-137-261419_review_history.pdf]

Native collagen VI delays early muscle stem cell differentiation

Samuele Metti, Francesco Da Ros, Giorgia Toniato, Matilde Cescon and Paolo Bonaldo

DOI: 10.1242/jcs.261419

Editor: Michael Way

Review timeline
Original submission: 17 June 2023
Editorial decision: 28 September 2023
First revision received: 4 January 2024
Accepted: 5 January 2024

Original submission

First decision letter

MS ID#: JOCES/2023/261419

MS TITLE: Native collagen VI counteracts myogenic differentiation and sustains stemness of adult muscle stem cells

AUTHORS: Samuele Metti, Francesco Da Ros, Giorgia Toniato, Matilde Cescon, and Paolo Bonaldo
ARTICLE TYPE: Short Report

We have now reached a decision on the above manuscript. It has taken much longer than I would like as we had difficulty finding reviewers over the summer period. In addition, the second reviewer did not provide a review even though they were reminded multiple times. Although far from ideal, given the delays I have decided to proceed with a single review.

To see the reviewers report and a copy of this decision letter, please go to: https://submit-jcs.biologists.organd click on the 'Manuscripts with Decisions' queue in the Author Area. (Corresponding author only has access to reviews.)

As you will see, the reviewer is positive but has raised a number of concerns and recommendations to strengthen the papers findings. I think the suggestions make sense and need to be addressed. If you can address their concerns I would be pleased to see a revised manuscript.

Please ensure that you clearly highlight all changes made in the revised manuscript. Please avoid using 'Tracked changes' in Word files as these are lost in PDF conversion.

I should be grateful if you would also provide a point-by-point response detailing how you have dealt with the points raised by the reviewers in the 'Response to Reviewers' box. Please attend to all of the reviewers' comments. If you do not agree with any of their criticisms or suggestions please explain clearly why this is so.

Reviewer 1

Advance summary and potential significance to field

© 2024. Published by The Company of Biologists under the terms of the Creative Commons Attribution License (https://creativecommons.org/licenses/by/4.0/).

This manuscript studies the role of type VI collagen in the regulation of muscle cell differentiation. Using myogenic cells, the authors show that native COLVI inhibits differentiation while preserving the characteristics of stem/progenitor cells. A putative effect of COLVI on the canonical Wnt pathway is suggested. To date, the main role of COLVI on muscle stem cells has been to contribute to the regulation of muscle stiffness. This study shows instead that COLVI may act as a ligand for a (as yet unidentified) receptor. Although the results are intriguing and present a new perspective on the role of COLVI in muscle stem cells, they are currently preliminary and require further experimental validation to reinforce their validity and significance.

Comments for the author

To enhance the scientific value of the manuscript, authors are encouraged to consider the following recommendations:

A key issue is to identify the receptor with which COLVI interacts. An attempt to do this was made using an inhibitory antibody against integrin b1, but no effect was detected. A control showing that the antibody works in the system is missing.
The authors could also use the RGDS peptide, a broad-spectrum integrin-binding competitor, to test the role of integrins more comprehensively. In addition, the tyrosine receptors DDR1 and DDR2 are known to bind collagens. Are these receptors expressed by C2C12 cells? If so, they should be targeted either with inhibitors (if available) or siRNA. Even if this series of experiments do not identify the receptor, they will reinforce the manuscript by excluding the known candidates.
It is also recommended to perform COLVI treatments on isolated myofibres in which satellite cells proliferate and self-renew, in order to validate the results in a more physiologically relevant context. It would also be interesting to carry out this experiment using Col6a1 knockout mice, which are available in the Bonaldo laboratory. Furthermore, using these KO mice, it would be interesting to describe the behaviour of Col6a1-/- muscle stem cells (proliferation, differentiation, Pax7 expression) and to perform rescue experiments with exogenous native COLVI.
Figure 2 describes the impact of FGF2 on MuSCs. Although the experiments are correctly performed, the results are not novel and probably do not warrant a full figure. Furthermore, although FGF2 is used as a reference in the following experiments, it is not clear how the FGF2 experiments are relevant to the topic of this study.
In Figure 3E, COLI, which is used as a control in the study, appears to have a significant effect on some differentiation markers. Instead, the authors state, "This conclusion was also supported by the expression of myogenic markers, which revealed significantly reduced levels of Myh3, Myog and Acta1 transcripts (Fig. 3E) and MyH3 and MyoG proteins (Fig. 3F) in Col6-treated cultures, but not in Col1-treated cultures." This conclusion does not seem to be accurate. Please discuss this in more detail.
In Fig. 4, a link with b-catenin is investigated. Nuclear b-catenin is not clear in the IFs provided. In fact, based on the IFs shown, it appears that COLI has a positive effect. Given the very low percentage of cells with nuclear b-catenin (Fig. 4a), it is recommended to use positive and negative control treatments to activate and block Wnt signaling, in order to check the staining and the experimental setup.

Minor comments:
Replace cell "roundness" measurements with "eccentricity" measurements.
Translated with www.DeepL.com/Translator (free version)

First revision

Author response to reviewers' comments

Reviewer #1

"This manuscript studies the role of type VI collagen in the regulation of muscle cell differentiation. Using myogenic cells, the authors show that native COLVI inhibits differentiation while preserving the characteristics of stem/progenitor cells. A putative effect

© 2024. Published by The Company of Biologists under the terms of the Creative Commons Attribution License (https://creativecommons.org/licenses/by/4.0/).

of COLVI on the canonical Wnt pathway is suggested. To date, the main role of COLVI on muscle stem cells has been to contribute to the regulation of muscle stiffness. This study shows instead that COLVI may act as a ligand for a (as yet unidentified) receptor. Although the results are intriguing and present a new perspective on the role of COLVI in muscle stem cells, they are currently preliminary and require further experimental validation to reinforce their validity and significance.

To enhance the scientific value of the manuscript, authors are encouraged to consider the following recommendations:"

We gratefully thank the Reviewer for the appreciation of our work and for the helpful comments and suggestions, allowing us to improve the quality of our work. In the revised manuscript, we carried out further experiments and added new data (new panel S2C, new Figure S3A-C, new panel S5A), also restructuring the main figures and moving some data in supplementary material (new panel S4B).

Please note that due to Editorial office requests and compliance with journal guidelines, the manuscript text has been extensively restructured and shortened, including a single "Results and discussion" section (instead of separate sections for Results and for Discussion). For the sake of clarity, changes in the text referring to new/amended results for the Reviewer are highlighted in red-colored fonts, whereas changes made in manuscript structure and length as per Editorial office requests are highlighted in blue-colored fonts.

"A key issue is to identify the receptor with which COLVI interacts. An attempt to do this was made using an inhibitory antibody against integrin b1, but no effect was detected. A control showing that the antibody works in the system is missing. The authors could also use the RGDS peptide, a broad-spectrum integrin-binding competitor, to test the role of integrins more comprehensively. In addition, the tyrosine receptors DDR1 and DDR2 are known to bind collagens. Are these receptors expressed by C2C12 cells? If so, they should be targeted either with inhibitors (if available) or siRNA. Even if this series of experiments do not identify the receptor, they will reinforce the manuscript by excluding the known candidates."

We thank the Reviewer for the useful comments, which led us to perform additional experiments as recommended.
First, to confirm that the beta1 integrin inhibitory antibody is indeed working in our in vitro system, and taking into account the crucial role of beta1 integrins in cell adhesion to ECM ligands, we treated C2C12 cells with the same anti-beta1 antibody and measured the time course of their adhesion capabilities to fibronectin, a major matrix ligand for beta1 integrins. As reported in the new Fig. S2C of the revised manuscript, treatment with the beta1 integrin inhibitory antibody showed a significantly delayed C2C12 cell adhesion to fibronectin when compared to control conditions, thus confirming the effective inhibitory activity of the antibody.
Additionally, starting from the suggestion of the Reviewer, we purchased several chemical compounds known to act as specific inhibitors of different classes of membrane receptors for ECM ligands, including several cell surface interactors of Col6, and measured the effects of Col6 on C2C12 differentiation in combination with each of the inhibitory compounds. These included broad-spectrum competitors of both RGD-dependent integrins (RGDS peptide and its negative control RGES) and RGD-independent integrins (TC-I 15, inhibitor of alpha2beta1, alpha1beta1 and alpha11beta1), as well as selective inhibitors of the tyrosine kinase receptors DDR1 and DDR2 (7rh and WRG-28, respectively). As reported in the completely new Figure S3 of the revised manuscript, none of these compounds displayed any distinct effect in counteracting the myogenic inhibition exerted by Col6 on differentiating C2C12 cells. As an unrelated finding of some interest for other studies, we only noted a direct effect of WRG28 (DDR2 inhibitor) in inhibiting C2C12 differentiation, but with no synergy with Col6 effects.
As rightly suggested by the Reviewer, although this set of experiments did not allow to identify the receptor involved in the anti-differentiating effects exerted by Col6 in myogenic cells, these new results allowed to rule out some candidates, excluding a major role of both integrin and non-integrin collagen receptors in mediating the effects displayed by soluble native Col6. In the revised manuscript, beside the presentation of the new data of Fig. S3 in the respective results section, we also added some sentences discussing these additional experiments and data.
We gratefully thank the Reviewer for the suggestions, which helped to reinforce our work and

© 2024. Published by The Company of Biologists under the terms of the Creative Commons Attribution License (https://creativecommons.org/licenses/by/4.0/).

conclusions.

“It is also recommended to perform COLVI treatments on isolated myofibres in which satellite cells proliferate and self-renew, in order to validate the results in a more physiologically relevant context. It would also be interesting to carry out this experiment using Col6a1 knockout mice, which are available in the Bonaldo laboratory. Furthermore, using these KO mice, it would be interesting to describe the behaviour of Col6a1-/- muscle stem cells (proliferation, differentiation, Pax7 expression) and to perform rescue experiments with exogenous native COLVI.”

Concerning the implementation of isolated myofibers for performing Col6 treatments and for validating our results on isolated MuSCs, we indeed had carefully considered this aspect but we chose to avoid it based on the concept that the usage of ex vivo myofibers is likely to mask the direct effect elicited by soluble Col6. Indeed, while past work from our team making use of ex vivo myofibers from WT and Col6 KO mice already showed that Col6 residing in the stem cell niche of myofibers has a key role in the modulation of MuSC behaviour, with a particular emphasis in the maintenance of mechanical properties and stiffness, our aim in the present work was to dissect the direct role exerted by soluble Col6 in the modulation of MuSC differentiation, without any interference imposed by other components.
It goes without saying that the Col6a1 null mouse is a highly valuable tool for dissecting the impact of Col6 ablation in muscle physiopathology. Concerning Col6a1 KO muscle stem cells, in past work we indeed showed that they display an abnormal behaviour in terms of in vivo differentiation and Pax7 expression and we demonstrated that such defects are relying upon altered mechanical properties with a distinctive lower stiffness caused by the lack of Col6 microfibrils in the endomysial ECM and in the muscle stem cell niche. As the aim of our present work was to investigate whether Col6 provided as a soluble extracellular ligand exerts any effect on cultured in vitro wild-type myogenic cells and MuSCs, we reasoned that a rescue of the altered satellite cell behaviour in ex vivo myofibers isolated from Col6a1 KO mice may be tough to obtain with such approach, since it also relies on changes in their matrix and niche stiffness and normalization of their biomechanical properties. In addition, difficulties in the interpretation of confusing or potentially negative results with such rescue experiments with ex vivo Col61 KO myofibers may be due to other changes imposed by the knockout condition, and not to a specific direct effect of soluble native Col6. For these reasons, in the present study we decided to focus on WT myogenic cells and MuSCs with the aim to investigate a potentially direct biochemical effect exerted by soluble Col6, independently on the known role of endomysial Col6 in regulating the ECM stiffness of satellite cell niche and without any possible influence imposed by the altered mechanical properties of Col6a1 KO muscles and ex vivo myofibers.

“Figure 2 describes the impact of FGF2 on MuSCs. Although the experiments are correctly performed, the results are not novel and probably do not warrant a full figure. Furthermore, although FGF2 is used as a reference in the following experiments, it is not clear how the FGF2 experiments are relevant to the topic of this study.”

We thank the Reviewer for the comment and suggestion. In the revised manuscript, we moved the panels of previous Fig. 2C to supplementary material (now Fig. S4B), considering their secondary relevance for manuscript progression. On the other hand, we chose to maintain the other panels of Fig. 2, as they allow both expert and non-expert readers to clearly grasp the experimental setup and differentiation protocol we used in this study, which in turn represent the basis for the experiments with MuSC and Col6 shown in the subsequent figures.

“In Figure 3E, COLI, which is used as a control in the study, appears to have a significant effect on some differentiation markers. Instead, the authors state, "This conclusion was also supported by the expression of myogenic markers, which revealed significantly reduced levels of Myh3, Myog and Acta1 transcripts (Fig. 3E) and MyH3 and MyoG proteins (Fig. 3F) in Col6-treated cultures, but not in Col1-treated cultures.” This conclusion does not seem to be accurate. Please discuss this in more detail.”

We thank the Reviewer for the comment. Indeed, differentiating MuSCs treated with Col1 displayed an opposite effect for some differentiation markers, with a significant upregulation of MyoG and MyoD transcript levels and a non-significant trend towards higher level of MyoG protein levels when

© 2024. Published by The Company of Biologists under the terms of the Creative Commons Attribution License (https://creativecommons.org/licenses/by/4.0/).

compared to untreated condition. Accordingly, in the revised manuscript we modified the presentation of these data and added one sentence describing more accurately the data obtained by Col1 treatment.

“In Fig. 4, a link with b-catenin is investigated. Nuclear b-catenin is not clear in the IFs provided. In fact, based on the IFs shown, it appears that COLI has a positive effect. Given the very low percentage of cells with nuclear b-catenin (Fig. 4a), it is recommended to use positive and negative control treatments to activate and block Wnt signaling, in order to check the staining and the experimental setup.”

We followed Reviewer’s recommendation and added new immunofluorescence data (Fig. S5A of the revised manuscript), showing that treatment with recombinant Wnt3a, used as a positive control, leads to nuclear localization of β-catenin in differentiating MuSCs, thus confirming the validity of the experimental setup and of beta-catenin immunostaining. We also changed the immunofluorescence panel of Fig. 4A, providing a better representative image of the untreated condition.

“Minor comments:
Replace cell "roundness" measurements with "eccentricity" measurements.”

We agree with the Reviewer of the confusing term “cell roundness” we inadvertently used. We have now amended both text and Fig. 3B, more appropriately referring to it as “circularity index” as we specify in the Materials and methods section and as defined in several literature studies [e.g., Eliazer, S., Muncie, J. M., Christensen, J., Sun, X., D’Urso, R. S., Weaver, V. M. and Brack, A. S. (2019). Wnt4 from the niche controls the mechano-properties and quiescent state of muscle stem cells. *Cell Stem Cell* **25**, 654-665].

Second decision letter

MS ID#: JOCES/2023/261419

MS TITLE: Native collagen VI counteracts myogenic differentiation and sustains stemness of adult muscle stem cells

AUTHORS: Samuele Metti, Francesco Da Ros, Giorgia Toniato, Matilde Cescon, and Paolo Bonaldo
ARTICLE TYPE: Short Report

I am happy to tell you that your manuscript has been accepted for publication in Journal of Cell Science, pending standard ethics checks.

© 2024. Published by The Company of Biologists under the terms of the Creative Commons Attribution License (https://creativecommons.org/licenses/by/4.0/).
